# The Peanut That Broke The Law

**Published:** 2024-09-20

**Authors:** Tad Brown

**Affiliations:** Department of History and Philosophy of Science, University of Cambridge

## Abstract

Crop surplus reduction programs from the New Deal had to contend with breeders’ efforts at state experiment stations to increase varietal yields. With peanuts, these countervailing forces were resolved by adjusting and eventually doing away with federal legislation.

The New Deal marked a fundamental recognition by the United States government that farmers were unable to earn a living without significant regulatory changes to commodity markets. Rural poverty and the threat of social unrest compelled federal intervention into agricultural capitalism. Agrarian reform during the Great Depression sought to address the impoverishment of farmlands and farmworkers.^1^ Toward this goal, New Deal policies from the 1930s aimed to influence commodity markets by curbing crop surplus. Yet while these government programs limited agricultural production, public breeders at state experiment stations sought to increase yields per acre. The scientific practice of crop breeding did not align with the New Deal agenda.

Crop varieties, as materials making the conditions of history, ought to be a primary concern within the framework of historical materialism. In *First the Seed*, Jack Kloppenburg identified a division of labor whereby public funds that sustained agricultural research ultimately delivered profits to private seed companies. Juxtapose this Marxist thesis by Kloppenburg with the argument of *Creating Abundance*, in which economic historians Alan Olmstead and Paul Rhode assert that crop breeding was a labor-saving technology that revolutionized U.S. agriculture as much as farm machinery before World War II.^2^ What emerges from these combined histories is the idea that varietal innovations reinforced the leverage of capital owners over labor. This is not to propose that public breeders intended to displace farmworkers or undermine New Deal policy. As I see it, progressive politics in the United States was bested by entrenched power structures and science as usual.

This article charts the historical trajectories of surplus management and breeding projects as they occurred with peanuts. I focus on peanuts because the New Deal program for the crop outlasted similar programs for all other crops save tobacco. With peanuts, federal limits on marketable production were contradicted by public breeders’ scientific pursuit of greater yields. The tension would eventually force a legislative change. As stated in the article’s title, a peanut variety broke the law as it was written.

## Part One: Discourage Surplus

Most historians agree that the Agricultural Adjustment Administration (AAA) was the most significant New Deal farm program to emerge from the crisis. A collective action plan, the AAA established price supports, acreage allotments, marketing agreements, and commodity loans to achieve parity between farmers’ incomes and income in other sectors. It is notable that the federal agenda to combat agricultural surplus was drafted during a time of national crisis. As one economist, a farmer during the Great Depression, later reflected: “The proposal was that we reduce production, turn inward, and create an American farm price structure basically higher than in the rest of the world.”^3^

Since its founding in the 1860s, the United States Department of Agriculture (USDA) has sought to educate the public about how to increase on-farm production. Direct government intervention in U.S agriculture can, however, be traced to World War I. A specialist from the Bureau of Markets outlined how, during the war, the Federal Reserve Board permitted banks to “make loans against warehouse receipts for potatoes when properly graded, packed, stored, and insured.”^4^ In September 1923, this infrastructure took on new meaning for peanuts when amendments to the 1916 Warehouse Act reclassified the peanut crop as “a storable product.”^5^ Graded peanuts in a licensed warehouse were as good as money in the bank.

Commodity crops experienced a downward price trend after World War I. The jarring slump threatened to undo the American peanut industry. Cotton producers faced similar depressed conditions and partly blamed peanuts for their woes. Peanuts, as a crop grown in the southern states, became a substitute for cottonseed in oil mills, especially following the arrival of the boll weevil.^6^ The pest had ravaged cotton fields across the southern region since the 1890s, doing injury to its agricultural economy. Given the fungibility of vegetable oils, mills could opt to purchase peanuts in lieu of cottonseed when commodity prices favored the former. Peanut farming gained traction in this environment.

Agricultural experts in the early twentieth century continued to debate how southern states might move away from cotton. Peanuts offered one possibility. The push for diversification, however, was frustrated by a lack of information. As it was, the USDA had no legal authority to gather statistics for commodity crops other than cotton and, soon thereafter, tobacco. A consolidated bill put before the House in 1931 authorized the Secretary of Agriculture to report on the quantity of peanuts in the hands of anyone in the industry—warehouses, threshers, pickers, crushers, brokers and dealers.^7^ Basically, it endeavoured to put the force of law behind the collection and sharing of information in the peanut industry.

Language for the proposed Peanut Statistics Bill largely copied that of the bill concerning tobacco. The two crops shared certain characteristics. As stated in the hearings, peanuts were not “seasonal things.”^8^ Warehouses could hold peanuts for years, as they did tobacco and cotton, and this durability within trade channels was a special advantage to processors with storage infrastructure. Congressmen who supported the Peanut Statistics Bill worried that the timing was far from ideal.^9^ The additional money required for reporting inventory was unlikely to be available due to the economic depression besetting the country. Indeed, monthly reporting on peanut statistics would not be required by law until 1936.^10^ Statistics appear to obtain greater importance in the USDA as part of the energetic New Deal initiative to limit surplus.

The Agricultural Adjustment Act of 1933, which created the AAA, marked the beginning of a comprehensive reformation of the industrial relations between crop growers and the government. The federal program included “a direct attempt to determine peanut prices” and then a compensatory tax on crop processing.^11^ This objective of boosting farmer prices was not independent of overseas trade. For instance, peanuts entering and leaving the domestic market influenced the balance of supply-and-demand as much as the peanuts pulled from American fields. The first head of the AAA actually advocated to control crop surplus through increased exports instead of production limits. His short-lived tenure reflected the low popularity of the proposal.^12^

On the topic of imports and exports, consider this report about the Revenue Act of 1934: “Perhaps the hardest fought provision of the entire Act of 1934 was the one having to do with taxes on coconut and allied oils…it was purely for protective purposes and was aimed primarily to check imports from the Philippines in order to aid American dairy interests.”^13^ Vegetable oils were a key ingredient in oleomargarine, a product that took market share from the domestic butter industry. President Franklin Roosevelt openly opposed the provision, however, as an insult to trade relations with the Philippines. Others challenged its alleged benefits for American farmers.^14^ Congress went ahead with a modified tariff. As it happened, the Virginia-Carolina region experienced a massive peanut crop in 1935, which, if not for slack in the oil market caused by the import taxes, would have doomed the peanut commodity price.^15^

In January 1936, the Supreme Court found the Agricultural Adjustment Act to be unconstitutional, and the tax on crop processing was terminated.^16^ Congress quickly followed with the Soil Conservation and Domestic Allotment Act to help national recovery, but this act lacked production controls. Granary stocks began to skyrocket. The 1937 Agricultural Marketing Agreement Act re-established federal authority to provide marketing orders. Finally, the Agricultural Adjustment Act of 1938 replaced the previous stop-gap measures. President Roosevelt authorized the “historic legislation” with its parity payments, marketing quotas, and federal crop insurance to end “the old familiar cycle of glut and scarcity.”^17^

The legislation of 1938 limited the production of six crops to improve farm commodity prices: wheat, corn, cotton, rice, tobacco, and peanuts. “The politically powerful basic crops provided only about 20% of agriculture’s income but received 75% of the program benefits,” recounted an economist.^18^ Under the program, regional associations bought peanuts directly from producers at fixed preferential prices.^19^ Excess peanuts, referred to as “additionals,” could only be sold for oil processing or export at lower market prices. These measures were conceived as temporary, yet many peanut growers in the United States became dependent on the federal price support. For decades to come, the government would stay involved in regulating the overproduction of peanuts under the USDA Fats and Oils Branch of the Commodity Credit Corporation (CCC).^20^

The most basic aspect of the reformed New Deal regulatory landscape for peanuts was the quota on production. Quotas governed how much land could be devoted to growing crops for the domestic market. Each allotment accorded by the peanut quota was expressed in terms of acreage so that peanuts were permitted to be grown only on specified amounts of land. Southern landowners had feared that the New Deal might upset their economic control over labor in the region. Yet because the quota system tied peanut allotments to land, the program upheld existing power dynamics.^21^ Each quota was effectively owned by the landowner. In the policy arena created by the New Deal, Congressmen from southern states ensured that federal subsidies guaranteed under the AAA program followed a pattern of rampant anti-Black discrimination. This is an example of how the racial governance of Jim Crow was extended through New Deal policies.^22^ Tenants and sharecroppers were excluded from federal farm programs through the discretionary controls of state and local committees.^23^ Instead, government checks and capital loans from the New Deal programs helped to finance agricultural mechanization, as landowners evicted sharecroppers and left plowshares to rust.

When the outbreak of World War II disrupted the international trade of fats and oils, the federal government showed a renewed enthusiasm for domestic peanuts.^24^ Congress authorized the Secretary of Agriculture in 1941 to raise price supports when necessary, in this case as part of the war effort. The Emergency Price Control Act of 1942 increased payment rates for designated commodities, including peanuts.^25^ This trend would continue well beyond World War II. The Agriculture Act of 1949 retained fixed price supports for two more years, only to be extended by lawmakers during the Korean War. Government purchasing of farm commodities encouraged production by ensuring high prices, even as the quota limited the acreage under cultivation. The dysfunctions of federalism were as familiar as the cycles of glut and scarcity. Crop science played its part.

## Part Two: Promote Yield

In 1931, B.B. Higgins, principal botanist at the Georgia Experiment Station, won federal funding under the Purnell Act to study the hybridization of peanuts.^26^ The Purnell Act tended to support research in agricultural economics or social science studies concerned with rural unrest.^27^ Higgins’ application took a different approach. In it, he proposed to “develop a high-yielding, disease-resistant, low-oil variety for hog feeding.”^28^ One-third of the Georgia peanut crop was hogged-off, a practice whereby hogs were run directly in the peanut fields to forage. At the time, the percentage of Georgia’s peanut harvest directed to hogging off was more than “all other states combined.”^29^ The practice of hogging-off peanuts reduced labor costs at harvest time, which explains why it had appeal. In addition, Higgins also asked for the Purnell funds to investigate the inheritance of disease resistance and other desirable traits because he believed that new varieties would benefit Georgia’s peanut supply.

By 1932, Higgins had a collection of “some 75 varieties and strains” under study and had obtained heredity records to assist in identifying “the more promising selections.”^30^ A systematic collection of peanut varieties furnished the Georgia Experiment Station with diverse breeding material. There are four main commercial peanut types in the U.S. (Virginia, Spanish, runners, Valencia), and each type consists of different varieties. Among the hundreds of experimental hybrids, Higgins crossed the varieties Spanish 18-38 and Basse—with Basse originating from the Gambia and Spanish 18-38 from Virginia—to produce the peanut strain known as GA 207.^31^

The breeding project led by Higgins resulted in several commercial types for the domestic market.^32^ In fact, popular peanut varieties in the United States across the twentieth century included GA 207 in their pedigree.^33^ Many of the peanuts selected early on at the Georgia Experiment Station, however, had disappointed food manufacturers. Higgins would later recall being asked by an industry representative for a peanut with high oil content, even if unsuitable as an edible peanut.^34^ The GA 207-3 (a line selection of the hybrid) appeared to satisfy this atypical request. It was “a tall, upright, bunch type with pods and seed slightly larger than those of Dixie Spanish,” a peanut variety previously selected by Higgins.^35^ Processors had rejected the GA 207-3 based on its insipid taste, but the kernels expressed a whopping 52–56 percent oil.^36^ As a crop to be cultivated for oil rather than food, these peanuts would have been exempt from the preferential pricing structure of the CCC.

As had its counterpart in Georgia, the Florida Experiment Station instituted a peanut breeding program based on experimental hybridization. Agronomist Fred Hull led the work in Gainesville, Florida, using federal funds from the Hatch Act to start the project. By 1930, the program in Gainesville had many peanut varieties under trial. One of Hull’s initial pursuits was to increase peanut foliage for hay production, but his main breeding objective became producing “a variety of Jumbo size with the high market quality of Spanish peanuts and the seed dormancy of the runner varieties.”^37^ The rest period in runner peanuts, also known as dormancy, allowed farmers to postpone harvesting till later in the season or to even hog-off a field in winter. Conversely, the lack of a dormancy period in Spanish peanuts caused severe losses with delayed harvest. Hull inferred that this tendency in Spanish peanuts could be corrected with a genetic contribution from runner peanuts.

Hull hybridized varieties from the two types, Spanish and runner, and then backcrossed the most dormant hybrid strains to Spanish parentage. The Florida Experiment Station had forty hybrids under study by 1931.^38^ A problem he encountered with these hybrids was that none were true breeding. Nonetheless, Hull continued to believe that selection would result in new stable varieties. The Department of Chemical Engineering at the University of Florida was, at the same time, doing experimental research on the “mechanism of oil formation” in peanuts.^39^ Hull would turn an eye towards the oil content of kernels as an indicator of varietal choice while looking for plants that would give Florida farmers an advantage.

In 1933, Hull and his colleague W.A. Carver (not to be confused with famed Black peanut scientist George Washington Carver) paced the sandy rows of breeding plots in Gainesville. Of the 200 peanut crosses sown that year, one exhibited the desired suite of “high yield, disease and insect resistance, and dormancy.”^40^ The Florida Experiment Station had managed to cross a Small White Spanish strain with Dixie Giant, a Jumbo peanut provided by the USDA.^41^ The next year, Hull and Carver planted 4,800 seeds from this hybrid.^42^ The cross showed exceptional promise. It outproduced other runners by upwards of thirty percent. Hull and Carver named it Dixie Runner and through cooperative efforts made seed available for planting in 1945.^43^ County-level crop improvement associations carried out the work of increasing Dixie Runner seed for regional distribution, as both peanut growers and processors instantly gravitated towards the variety.

Peanut breeding at agricultural experiment stations in the southern United States during the 1930s ran contrary to the New Deal policy agenda of crop reduction. Public breeders intended to increase yield while federal legislation curtailed it. Of course, the AAA contained its own contradictory measures. Meant to help struggling farmers, federal programs privileged a class of large-scale white landowners who benefited from direct government subsidies.^44^ All the while, land consolidation continued to remove farmers from American agriculture. Peanut breeding fed into these economic relations by supplying quota owners with high-yielding varieties that suited the demands of food processors.

In 1943, Hull and Carver included hybrid selections from the Georgia Experiment Station in the varietal studies in Gainesville.^45^ Despite being rejected in Georgia for its flavor profile, the GA 207-3 attracted the interest of the Florida breeding program due to its yield and oil content. Ten years later, in August 1953, the Florida Agricultural Experiment Station issued a new peanut variety named Florispan Runner.^46^ The variety originated from a cross between Georgia 207-3 and the parentage of Dixie Runner. As the name suggests, Florispan Runner had a running growth-habit with seeds resembling Spanish-type peanuts. The new peanut exhibited a key advantage over the Dixie Runner. Carver described the seeds as “full, smooth and fairly free of the wrinkles and grooves that make Dixie Runner and Florida Runner hard to blanch,” a quality in the nut that was considered an imperative for the edible trade.^47^ Again, if farmers were to sell their peanut harvest to domestic food manufacturers, the variety needed to conform to their liking.

Continued breeding in Gainesville led to the release of Florunner in 1969.^48^ As one historian of peanut butter has stated, when “Florunner came on the scene, there was a significant improvement in the oil chemistry and flavor” of its nuts, a result of decades of research.^49^ The nut had a higher percentage of oleic acid, which made it good for shelf-stable peanut butter. It also had a better shelling rate, or more peanuts per ton, which manufacturers desired. Most importantly, the variety was super prolific with yields upwards of twenty-five percent higher than that of its predecessors. Within five years, farmers in the southern states grew Florunner on ninety percent of their peanut acreage.^50^ Price supports, big yields, and international tariffs on peanut butter guaranteed the value of the bulk domestic crop.^51^ Peanut scientists observed the trend with a sense of wonder. One noted that “[a]s fast as [Florunner] seed became available, a profit-taking society polarized towards its monocultural production.”^52^

## Part Three: End Quota

From 1950 to 1980, the allotted peanut acreage in the United States held relatively stable, yet yield per acre tripled.^53^ Research at the state experiment stations was largely responsible, especially breeding and especially Florunner. It took a decade for the peanut variety to expose the limitations of controlling commodity surplus by limiting the acreage planted. Then, Florunner broke the law. Nothing about the variety was illegal, but its high yields led Congress to redraft legislation from the New Deal. The government revised the peanut quota in 1981, capping how much each allotment could sell (in pounds) rather than how much land could be farmed (in acres).^54^ In other words, the historic achievements of peanut breeding altered the terms of federal regulation.

Another important detail about the updated legislation is that it permitted “quota peanuts to be produced on the property of the renter” rather than the landowner, so long as the quota stayed within the county where it originated.^55^ Freeing the peanut quota from being tied to specific acreage had the potential to disrupt power relations between landowners and those who leased land to farm peanuts. Not much would change, however: ownership of a quota allotment is what mattered for deriving rents from the quota. As before, surplus peanuts were sold at market price and mostly to export or crush for oil.^56^ Old trade restrictions limiting peanut imports into the United States now faced the possibility of retaliatory measures abroad.

By the 1990s, the New Deal peanut commodity program was costing taxpayers money because the CCC was paying to stockpile surplus peanuts. The federal government, struggling to find a commercial market at the inflated price, lowered its price support in the 1996 Farm Bill.^57^ Representatives in southern states fought to protect the measure until the end. Congress terminated the quota altogether in 2002. No longer would peanut production be subject to quotas by the acre or the pound. The government decided to initiate a price-triggered payment support for peanuts as was commonplace for other crops.^58^ (Only if the commodity market dipped below a set price would the government help producers.) In a final act of profligacy, the USDA compensated those who lost a peanut quota with a one-off payment.

The end of the quota revealed a pattern that had been underway since its inception. Government limits on production were working against the scientific drive for a bonanza harvest. In time, the yield gains of peanut varieties bred at state experiment stations overwhelmed federal regulation to buoy the crop commodity price. Whose interests were served in the process? Because of how quotas were allotted and how peanuts were bred, large landowners and industrial food processors captured the profits. Everybody else got cheap, shelf-stable peanut butter.

The history of peanuts in the United States demonstrates an interplay of science and politics. If there are lessons to learn from adopting a materialist perspective of this history, one lesson would have to concern the influence of agricultural breeding on the conditions of social change. Crop varieties yield more than more crops. Two historians concluded their study of the New Deal with this recognition: “The art of politics, as southerners have so aptly demonstrated in Congress, remains the essence of statecraft—not scientific management.”^59^ The legacy of the New Deal with respect to peanuts calls for revision to their statement. To conclude, I submit my own: The politics of managing science are essential to the art of statecraft.

## Footnotes

1 Jess Gilbert, *Planning Democracy: Agrarian Intellectuals and the Intended New Deal* (Yale University Press, 2015).

2 Jack Ralph Kloppenburg Jr, *First the Seed: The Political Economy of Plant Biotechnology*, Second Edition (University of Wisconsin Press, 2005); Alan L. Olmstead and Paul W. Rhode, *Creating Abundance: Biological Innovation and American Agricultural Development* (Cambridge University Press, 2008).

3 Don Paarlberg, “Effects of New Deal Farm Programs on the Agricultural Agenda a Half Century Later and Prospect for the Future,” *American Journal of Agricultural Economics* 65, no. 5 (1983): 1163-1167, 1163-4.

4 Harold W. Samson, “The March of Standardization,” In *Yearbook of Agriculture, 1920* (USDA, 1921), 353.

5 Henry Johnson Clay, *Marketing Peanuts and Peanut Products* (USDA, 1941), 20.

6 ”South Will Raise Seventy Million Bushels of Peanuts,” *The Montgomery Advertiser*, Montgomery, July 20 1917, 9.

7 The Senate Bill (S 3409), drafted by Georgia Senator Walter George, was originally introduced in 1930. The House Bill (HR 10345) authored by a representative from Virginia, included a broader number of occupations who were required to provision the Secretary with information. *Collection and Publication of Peanut Statistics* (United States 71^st^ Congress, 3d Session, Report No. 2411, January 28, 1931).

8 Ibid.

9 Letter from Senator Walter F. George to Bob Barry, March 25, 1932 (Emory University Archives, MSS 8, Box 107, Folder 1); Letter from Senator C.R. Crisp to Bob Barry, March 26, 1932 (Emory University Archives, MSS 8, Box 107, Folder 1).

10 *Peanut Statistics*, 7 USC § 951-958 (1936).

11 Richard H. Leach, “The Federal Government and the Peanut Industry,” *Southern Economic Journal* 211 (1954): 53-61, 54.

12 Wayne D. Rasmussen, “New Deal Agricultural Policies After Fifty Years,” *Minnesota Law Review* 68, no. 2 (January 1984): 353-378.

13 Roy G. Blakey and Gladys C. Blakey, “The Revenue Act of 1934,” *The American Economic Review* 24, no. 3 (1934): 450-46, 458.

14 Philip G. Wright, “Effects on American Agriculture of a Duty on Philippine Coconut Oil and Copra,” *Journal of Farm Economics* 14, no. 2 (1932): 311-318.

15 ”Suffolk Storage Facilities are Open to Growers,” *The Suffolk News-Herald*, November 19, 1935, 1.

16 *U.S. v Butler*, 297 US 1 (1936).

17 Franklin D. Roosevelt, Statement on Signing the Agricultural Adjustment Act of 1938. Online by Gerhard Peters and John T. Woolley, The American Presidency Project at: https://www.presidency.ucsb.edu/node/209459

18 D. Paarlberg, “Effects of New Deal Farm Programs on the Agricultural Agenda a Half Century Later and Prospect for the Future,” *American Journal of Agricultural Economics* 65, no. 5 (1983): 1163-1167, 1164.

19 Clay, *Marketing Peanuts*, p. 31.

20 Richard H. Leach, “The Federal Government and the Peanut Industry,” *Southern Economic Journal* 211 (1954): 53-61; James Pease, John Lehman, and David Orden, “Proposed End of Peanut Quota Program: Economic Effects on Virginia Producers,” *Virginia Tech Newsletter* (November 2001): 1-19.

21 Jack Temple Kirby, “The Transformation of Southern Plantations c. 1920 – 1960,” *Agricultural History* 57, no. 3 (1983): 257-276.

22 Kimberley S. Johnson, “Racial Orders, Congress, and the Agricultural Welfare State, 1865-1940,” *Studies in American Political Development* 25 (2011): 143-161.

23 Pete Daniel, “The Transformation of the Rural South: 1930 to the Present,” *Agricultural History* 55, no. 3 (1981): 231-248.

24 Bennett S. White, Jr. and Edith T. Denhardt, “Chronic Surpluses of Agricultural Commodities in the Post-War Period,”*Journal of Farm Economics* 25, no. 4 (1943): 743-758.

25 Edward L. Schapsmeier and Frederick H. Schapsmeier, “Farm Policy from FDR to Eisenhower: Southern Democrats and the Politics of Agriculture,” *Agricultural History* 53, no. 1 (1979): 352-371, 361.

26 B.B. Higgins, *Purnell Fund Investigation #7* (University of Georgia, UA 07-022, Box 19, Folder 22, 1932).

27 Joel P. Kunze, “The Purnell Act and Agricultural Economics,” *Agricultural History* 62, no. 2 (1988): 131-149.

28 Higgins, *Purnell Fund*.

29 B.B. Higgins, “New Types of Peanuts and Their Uses as Feed and as Cash Crops,” (Media Release, WSB Dixie Farm Hour, August 23, 1940) UGA Griffin Campus Library, 1.

30 Higgins, *Purnell Fund*.

31 B.B. Higgins, K.T. Holley, T.A. Pickett, and C.D. Wheeler, *Peanut Breeding* (Georgia Agricultural Experiment Station Bulletin No. 213, June 1941).

32 Everett S. Luttrell, Richard T. Hanlin, Donald R. Sumner, and Jerry T. Walker, *A Collective Account of the History of the Department of Plant Pathology at The University of Georgia (Origins to 1999)* (University of Georgia, 2000).

33 T.G. Isleib and J.C. Wynne, “Use of Plant Introductions in Peanut Improvement.” In H. Shands and L.E. Weisner (eds), *Use of Plant Introductions in Cultivar Development, Part 2* (Crop Science Society of America, 1992), 75-116.

34 B.B. Higgins and Wallace K. Bailey, *New Varieties and Selected Strains of Peanuts* (Georgia Agricultural Experiment Station Bulletin No. 11, November 1955).

35 Ibid, 29.

36 Ibid, 29-30.

37 *Annual Report of the Florida Agricultural Experiment Station for the year 1932* (Florida Experiment Station, 1933), 30.

38 *Annual Report of the Florida Experiment Station for the year 1931* (Florida Experiment Station, 1932).

39 Letter from Laurence M. Emanuel to G.W. Carver, November 6, 1933 (Tuskegee University Archives, George Washington Carver Papers, Collection 006, Box 023, Folder 3).

40 *Annual Report of the Florida Experiment Station for the year 1934* (Florida Experiment Station, 1935), 26.

41 Ibid.

42 *Annual Report of the Florida Experiment Station for the year 1935* (Florida Experiment Station, 1936), 36.

43 *Annual Report of the Florida Experiment Station for the year 1944* (Florida Experiment Station, 1945), 34.

44 See Debra A. Reid, “African Americans and Land Loss in Texas: Government Duplicity and Discrimination Based on Race and Class,” *Agricultural History* 77, no. 2 (2003): 258-292.

45 *Annual Report of the Florida Experiment Station for the year 1944* (Florida Experiment Station, 1945), 37.

46 W.A. Carver, “The Florispan Runner Peanut Variety,” (Florida Agricultural Experiment Station Circular S-62, 1953).

47 Ibid.

48 A.J. Norden, R.W. Lipscomb, and W.A. Carver, “Florunner–A New Peanut Variety,” (Florida Agricultural Experiment Station Circular S-196, 1969), 3-14.

49 Jon Krampner, *Creamy and Crunchy: An Informal History of Peanut Butter, the All-American Food* (Columbia University Press, 2014), 160.

50 Ray O. Hammons, “Peanuts: Genetic Vulnerability and Breeding Strategy,” *Crop Science* 16 (1976): 527-530.

51 Robert B. Borges, “Trade and the Political Economy of Agricultural Policy: The Case of the United States Peanut Program,” *Journal of Agricultural and Applied Economics*, 27, no. 2 (1995): 595-612.

52 Ray O. Hammons, “Genetic Vulnerability in Peanuts: A Second Look,” (pp. 17-20) *Proceedings of the American Peanut Research and Education Association, Inc*. 6, no. 1 (August, 1974), 17.

53 Borges, “Trade and the Political Economy,” 600.

54 James Johnson, Richard W. Rizzi, Sara D. Short, and R. Thomas Fulton, *Provision of the Agriculture and Food Act of 1981* (Economic Research Service. Staff Report No. AGES811228, January 1982).

55 Pease et al., “Proposed End,” 3.

56 Patrick Archer, “Overview of the Peanut Industry Supply Chain.” In R.O. Hammons, D. Herman and H. T. Stalker (eds), *Peanuts* (AOCS Press, 2016), 253-266.

57 Pease et al., “Proposed End,” 1.

58 Dennis A. Shields, *U.S. Peanut Program and Issues*. Congressional Research Service 7-5700 (19 August 2015).

59 Schapsmeier and Schapsmeier, “Farm Policy from FDR to Eisenhower,” 371.

